# Breast and gynecological cancers in Croatia, 1988-2008

**DOI:** 10.3325/cmj.2012.53.100

**Published:** 2012-04

**Authors:** Iva Kelava, Karlo Tomičić, Marina Kokić, Ante Ćorušić, Pavao Planinić, Iva Kirac, Jure Murgić, Tomislav Kuliš, Ariana Znaor

**Affiliations:** 1 Fran Mihaljević University Hospital for Infectious Diseases, Zagreb, Croatia; 2 Department of Gynecology and Obstetrics, University Hospital Center Zagreb, Zagreb, Croatia; 3 Health Centre Zagreb (East), Zagreb, Croatia; 4 Sestre Milosrdnice University Hospital Center, Zagreb, Croatia; 5 Department of Urology, University Hospital Center Zagreb, University of Zagreb School of Medicine, Zagreb, Croatia; 6 Andrija Štampar School of Public Health, University of Zagreb School of Medicine, Zagreb, Croatia; 7 Croatian National Cancer Registry, Croatian National Institute of Public Health, Zagreb, Croatia; * The first two authors contributed equally to this article.

## Abstract

**Aim:**

To analyze and interpret incidence and mortality trends of breast and ovarian cancers and incidence trends of cervical and endometrial cancers in Croatia for the period 1988-2008.

**Methods:**

Incidence data were obtained from the Croatian National Cancer Registry. The mortality data were obtained from the World Health Organization (WHO) mortality database. Trends of incidence and mortality were analyzed by joinpoint regression analysis.

**Results:**

Joinpoint analysis showed an increase in the incidence of breast cancer with estimated annual percent of change (EAPC) of 2.6% (95% confidence interval [CI], 1.9 to 3.4). The mortality rate was stable, with the EAPC of 0.3% (95% CI, -0.6 to 0.0). Endometrial cancer showed an increasing incidence trend, with EAPC of 0.8% (95% CI, 0.2 to 1.4), while cervical cancer showed a decreasing incidence trend, with EAPC of -1.0 (95% CI, -1.6 to -0.4). Ovarian cancer incidence showed three trends, but the average annual percent change (AAPC) for the overall period was not significant, with a stable trend of 0.1%. Ovarian cancer mortality was increasing since 1992, with EAPC of 1.2% (95% CI, 0.4 to 1.9), while the trend for overall period was stable with AAPC 0.1%.

**Conclusion:**

Incidence trends of breast, endometrial, and ovarian cancers in Croatia 1988-2008 are similar to the trends observed in most of the European countries, while the modest decline in cervical cancer incidence and lack of decline in breast cancer mortality suggest suboptimal cancer prevention and control.

Breast and gynecological cancers are among the seven most common female cancers in Croatia: in 2008 breast cancer was the most common cancer with the proportion of 26% of all cancer sites, endometrial cancer ranked fourth (6%), ovarian cancer (with fallopian tubes cancer) sixth (5%), and cervical cancer seventh (4%) (1).

Breast, endometrial, and ovarian cancers share some similar risk factors like early menarche, late menopause, obesity, and low parity (2-5). Also, breast cancer in personal history increases the risk of endometrial and ovarian cancer (6). Delayed childbearing increases the risk of breast cancer but seems to have no impact on the development of ovarian and endometrial cancer (3-5). Diabetes mellitus increases the risk of endometrial and breast cancer (7,8). Use of tamoxifen or other selective estrogen receptor modulators increases the risk of endometrial and ovarian cancer, while the use of combined oral contraceptives is a protective factor (2,9,10). Also, tobacco smoking and alcohol intake reduce the risk of endometrial cancer (2,11,12). Alcohol intake and both oral contraceptives and hormonal replacement therapy are risk factors for breast cancer (2,13,14). Multiparty and physical activity are protective factors for all three cancers (2,4,15,16). Low socioeconomic status, sexually transmitted diseases, promiscuity, unprotected sexual behavior, earlier age of first intercourse, and smoking are risk factors for cervical cancer (2,17-23). Infection with human papillomavirus is considered as a necessary cause of cervical cancer (24).

The aim of this study was to report the incidence and mortality of breast and ovarian cancers and incidence of endometrial and cervical cancers, analyze the trends in the period 1988-2008, and compare them to other European countries.

## Materials and methods

### Data sources

Incidence data for the period 1988-2008 were obtained from the Croatian National Cancer Registry. The Registry, founded in 1959, covers the whole Croatian population (approximately 4.4 million persons) and relies on mandatory cancer notifications from primary and secondary health care sources and death certificates from the Croatian Bureau of Statistics. The Registry contributed data to the last three volumes of the Cancer Incidence in Five Continents series (25-27). Breast cancer was classified in the International Classification of Diseases as ICD-10 C50 and ICD-9 174, endometrial cancer as ICD-10 C54 and ICD-9 182, cervical cancer as ICD-10 C53 and ICD-9 180, and ovarian cancer as ICD-10 C56 and ICD-9 183. More than 95% of uterine corpus cancers are endometrial so we referred to all of them as endometrial cancer. The number of breast and ovarian cancer deaths were obtained from the World Health Organization (WHO) mortality database (28). Due to the frequent assignment of “malignant neoplasm of uterus, part unspecified” (ICD-10 C55) as the underlying cause of death in the analyzed period, and consequent underestimation of cervical and endometrial cancer mortality, we did not include these data in the analysis (28).

### Statistical analysis

Age-standardized rates (ASR) of cancer incidence and mortality were calculated by the direct standardization method, using the world standard population as a reference (29). To calculate age-specific incidence and mortality rates for breast cancer, we used the United Nations population estimates (30).

To describe incidence and mortality trends by calendar period, we carried out joinpoint regression analysis using the software Joinpoint Regression Program, Version 3.5.2, October 2011 (31). The analysis included the logarithmic transformation of the rates, standard error, maximum number of five joinpoints, and minimum of four years between two joinpoints. All other program parameters were set to default values. The aim of the approach is to identify possible joinpoints where a significant change in the trend occurs. The method identifies joinpoints based on regression models with 0-5 joinpoints. The final model selected was the most parsimonious of these, with the estimated annual percent change (EAPC) based on the trend within each segment (31). To quantify the trend over a fixed number of the years, the average annual percent change (AAPC) was calculated. The AAPC is computed as a geometric weighted average of the EAPC trend analysis, with the weights equal to the lengths of each segment during the prespecified fixed interval.

In describing trends, the terms “significant increase” or “significant decrease” signify that the slope of the trend was statistically significant (*P* < 0.05). For non-significant trends (*P* > 0.05), we used the terms “stable” (for EAPC between -0.5% and 0.5%), “non-statistically significant increase” (for EAPC>0.5%), and “non-statistically significant decrease” (for EAPC<-0.5%). All statistical tests were two sided.

## Results

### Breast cancer

From 1988 to 2008, the number of new breast cancer cases increased from 1220 to 2472 and ASR of incidence increased from 35.5 to 61.9 per 100 000. The number of deaths increased from 670 to 902, while ASRs remained stable (Table 1).

**Table 1:** Breast cancer incidence and mortality data, Croatia 1988-2008. Number of cases, crude rate, and age standardized rate (ASR) per 100 000

Year	Incidence			Mortality		
	N	crude rate	ASR	N	crude rate	ASR
**1988**	1220	52.7	35.5	670	28.9	18.0
**1989**	1353	58.3	38.8	661	28.5	17.8
**1990**	1461	62.7	40.7	710	30.5	18.4
**1991**	1351	57.5	37.9	758	32.3	19.8
**1992**	1429	60.3	37.7	730	30.8	18.0
**1993**	1539	64.3	40.6	758	31.7	18.4
**1994**	1658	68.7	43.6	749	31.1	17.7
**1995**	1779	73.6	45.4	768	31.8	18.0
**1996**	1782	73.8	45.8	706	29.3	16.9
**1997**	1883	78.6	47.7	769	32.1	17.8
**1998**	1908	80.3	47.8	794	33.4	18.4
**1999**	2544	108.1	67.4	856	36.4	19.7
**2000**	2038	87.3	51.7	843	36.1	18.5
**2001**	2233	96.1	57.0	832	35.8	18.5
**2002**	2052	88.6	51.1	804	34.7	17.1
**2003**	2251	97.4	55.8	822	35.6	16.9
**2004**	2164	93.8	52.8	825	35.8	17.9
**2005**	2303	100.0	56.2	922	40.0	18.0
**2006**	2205	95.9	54.3	830	36.1	17.1
**2007**	2574	112.1	62.7	861	37.5	17.3
**2008**	2472	107.9	61.9	902	39.4	17.6

**Figure 1:**
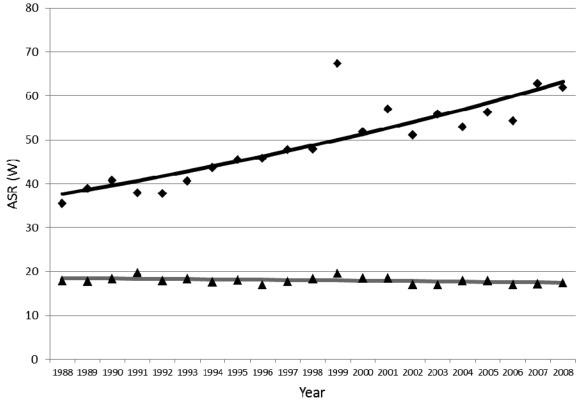
Joinpoint analysis of incidence and mortality of breast cancer in Croatia, 1988-2008. Rhomb – incidence; triangle – mortality; ASR (**W**) – age-standardized rate (using world standard population).

Joinpoint analysis of incidence showed a significantly increasing trend, with EAPC of 2.6% (95% confidence interval [CI], 1.9 to 3.4). Mortality trend was stable, with EAPC of -0.3% (95% CI, -0.6 to 0.0) (Table 2, Figure 1). When analyzed by 10-year age groups (Table 2), the incidence was increasing in all age groups. A significant change in the incidence trend was most pronounced in 2005 in the 60-69 age group, with EAPC of 14.2% in the period 2005-2008. Mortality decreased in younger age groups (30-49 years) and increased in older age groups (>60 years). In the age group 50-59 years, there was a change in trend starting from 1999. In that age group, mortality was increasing (EAPC 1.7%) from 1988 to 1999, when it started to decrease (EAPC -3.5%).

**Table 2:** Joinpoint analysis of breast cancer incidence and mortality in Croatia, 1988-2008 with the estimated annual percent change (EAPC) and 95% confidence intervals (CI)

	Trend 1 (years)	EAPC (95% CI)	Trend 2 (years)	EAPC (95% CI)
**Incidence by age-group:**				
30-39	1988-2008	1.1 (-0.5 to 2.8)		
40-49	1988-2008	1.7* (0.6 to 2.7)		
50-59	1988-1999	6.2* (4.9 to 7.6)	1999-2008	0 (-1.6 to 1.8)
60-69	1988-2005	3.0* (2.2 to 3.9)	2005-2008	14.2* (1.7 to 28.3)
70-79	1988-1999	3.5* (2.4 to 4.7)	1999-2008	-0.1 (-1.7 to 1.4)
>80	1988-2003	2.7* (1.5 to 3.8)	2003-2008	-3.4 (-8.9 to 2.3)
Overall	1988-2008	2.6* (1.9 to 3.4)		
**Mortality by age-group:**				
30-39	1988-2008	-3.2* (-4.8 to -1.5)		
40-49	1988-2008	-1.5* (-2.5 to -0.5)		
50-59	1988-1999	1.7* (0.4 to 3.1)	1999-2008	-3.5* (-5.3 to -1.7)
60-69	1988-2008	0.3 (-0.3 to 0.9)		
70-79	1988-2008	0.2 (-0.5 to 1)		
>80	1988-2008	1.6* (0.9 to 2.3)		
Overall	1988-2008	-0.3 (-0.6 to 0)		

*Statistically significant trend.

### Endometrial cancer

**Figure 2:**
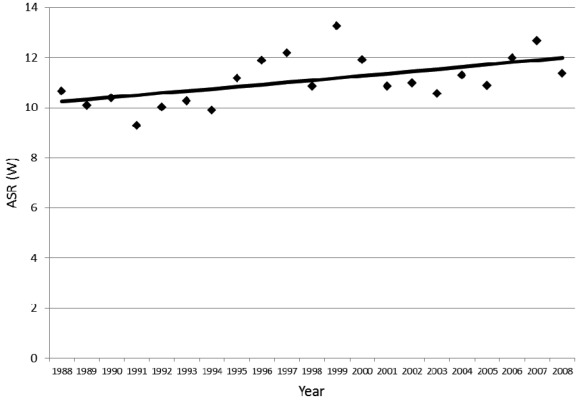
Joinpoint analysis of incidence of endometrial cancer in Croatia, 1988-2008. ASR (**W**) – age-standardized rate (using world standard population).

In 1988 there were 390, and in 2008 520 new cases of endometrial cancer. The ASRs increased from 10.7/100 000 in 1988 to 11.4/100 000 in 2008 (Table 3). Joinpoint analysis revealed a constant and significant increase, with the EAPC of 0.8% (95% CI, 0.2 to 1.4) for the period of 20 years (Table 4, Figure 2).

**Table 3:** Gynecological cancer incidence and mortality data, Croatia 1988-2008. Number of cases, crude rate, and age standardized rate (ASR) per 100 000

Year	Cervical cancer incidence			Endometrial cancer incidence			Ovarian cancer incidence			Ovarian cancer mortality		
	N	crude rate	ASR	N	crude rate	ASR	N	crude rate	ASR	N	crude rate	ASR
**1988**	406	17.5	12.2	390	16.8	10.7	337	14.6	9.8	230	9.93	6.60
**1989**	429	18.5	13.3	382	16.5	10.1	372	16.0	10.8	220	9.48	5.87
**1990**	399	17.1	11.9	391	16.8	10.4	393	16.9	11.3	239	10.25	6.28
**1991**	340	14.5	10.3	357	15.2	9.3	361	15.4	9.9	227	9.66	5.65
**1992**	375	15.8	11.0	389	16.4	10.0	390	16.4	10.6	205	8.64	5.31
**1993**	415	17.3	12.5	403	16.8	10.3	397	16.6	10.8	243	10.15	5.95
**1994**	405	16.8	12.0	414	17.2	9.9	383	15.9	10.7	229	9.49	5.86
**1995**	416	17.2	11.9	469	19.4	11.2	427	17.7	11.1	212	8.76	5.01
**1996**	365	15.1	10.5	511	21.2	11.9	418	17.3	10.5	239	9.90	5.55
**1997**	394	16.4	11.2	528	22.0	12.2	419	17.5	10.9	241	10.05	5.56
**1998**	358	15.1	10.2	475	20.0	10.9	426	17.9	10.7	255	10.74	5.88
**1999**	403	17.1	11.4	578	24.6	13.3	537	22.8	13.8	279	11.86	6.27
**2000**	426	18.2	12.7	508	21.8	11.9	550	23.6	14.5	267	11.43	6.01
**2001**	320	13.8	9.7	477	20.5	10.8	516	22.2	13.1	311	13.39	6.90
**2002**	363	15.7	10.7	488	21.1	11.0	489	21.1	12.1	289	12.48	6.37
**2003**	314	13.6	9.1	471	20.4	10.6	466	20.2	11.7	275	11.90	5.97
**2004**	330	14.3	9.8	509	22.1	11.3	432	18.7	10.7	278	12.05	6.15
**2005**	320	13.9	9.4	479	20.8	10.9	490	21.3	12.2	287	12.46	5.94
**2006**	345	15.0	10.4	534	23.2	12.0	450	19.6	11.1	291	12.66	6.16
**2007**	387	16.9	11.3	572	24.9	12.7	468	20.4	12.0	299	13.03	6.42
**2008**	359	15.7	10.2	520	22.7	11.4	439	19.2	10.5	326	14.23	7.09

**Table 4:** Joinpoint analyses of gynecological cancers incidence and mortality with the estimated annual percent change (EAPC) and 95% confidence intervals (CI) in Croatia, 1988-2008

Trend segment	Lower endpoint	Upper endpoint	EAPC (95% CI)
**Cervical cancer incidence**			
1	1988	2008	-1.0* (-1.6 to -0.4)
**Endometrial cancer incidence**			
1	1988	2008	0.8* (0.2 to 1.4)
**Ovarian cancer incidence**			
1	1988	1997	0.4 (-1.2 to 1.9)
2	1997	2000	8.1 (-7.4 to 26.2)
3	2000	2008	-3.1* (-4.8 to -1.4)
**Ovarian cancer mortality**			
1	1988	1992	-4.1 (-10.2 to 2.3)
2	1992	2008	1.2* (0.4 to 1.9)

*Statistically significant trend.

### Ovarian cancer

**Figure 3:**
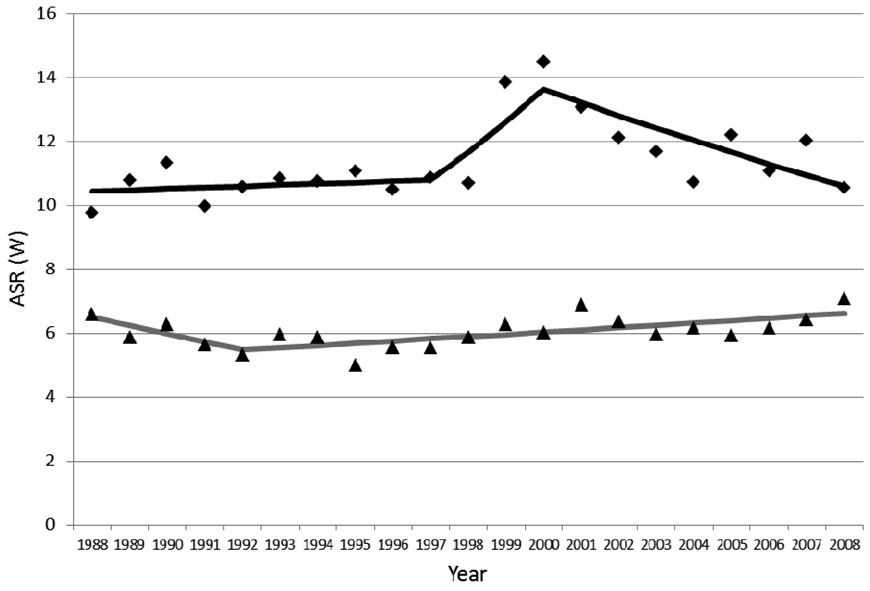
Joinpoint analysis of incidence and mortality of ovarian cancer in Croatia, 1988-2008. Rhomb – incidence; triangle – mortality; ASR (**W**) – age-standardized rate (using world standard population).

The number of new ovarian cancer cases increased from 337 in 1988 to 439 in 2008 (Table 3). Trend analysis of ovarian cancer incidence showed three trends (Figure 3, Table 4). Changes in the trend occurred in 1997 and 2000. Between 1988 and 1997, the trend was stable, with EAPC 0.4% (95% CI, -1.2 to 1.9). Between 1997 and 2000, there was a non-significant increase in incidence, with EAPC 8.1% (95% CI, -7.4 to 26.2). Between 2000 and 2008, there was a significant decreasing incidence, with EAPC -3.1% (95% CI, -4.8 to -1.4) (Table 4). AAPC for the overall period was not significant, with a stable trend of 0.1% (95% CI, -2.2 to 2.4).

Joinpoint analysis of ovarian cancer mortality revealed two trends. Trend 1 showed a non-significant decrease, with EAPC -4.12% (95% CI, -10.2 to 2.3). Trend 2 showed a significant increase of EAPC 1.2% (95% CI, 0.4 to 1.2%). AAPC for the overall period was not significant, with a stable trend of 0.1% (95% CI, -1.2 to 1.4).

### Cervical cancer

**Figure 4:**
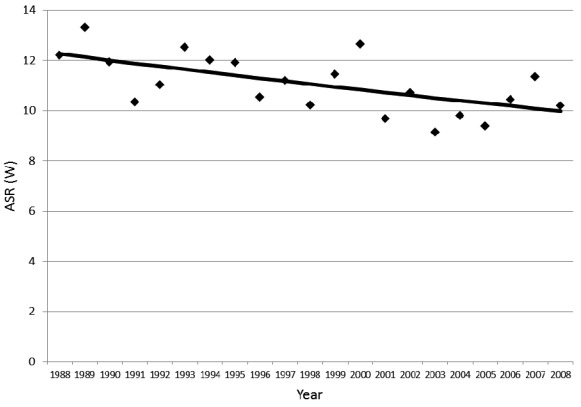
Joinpoint analysis of incidence of cervical cancer in Croatia, 1988-2008. ASR (**W**) – age-standardized rate (using world standard population).

In the period 1988-2008, the number of new cervical cancer cases decreased from 406 to 359, or with ASR of 16% – from 12.2/100 000 to 10.2/100,000 (Table 3). Joinpoint trend analysis showed a decrease in the incidence with a significant annual change of -1.0%, (95% CI, -1.6 to -0.4) (Table 4, Figure 4).

## Discussion

### Breast cancer

According to our results and GLOBOCAN estimates for 2008 (32), Croatia is among the European countries with an intermediate breast cancer incidence and mortality. The increasing incidence trend in Croatia 1988-2008 is comparable to trends in other European countries. Thirty-five countries with Caucasian majority population showed an increase in the incidence of breast cancer in 1990-2002 period, particularly in Central and Eastern European countries. The increase was slightly higher in the countries with initially lower incidence (33).

Possible explanations for the increase in the incidence in Croatia are a higher prevalence of lifestyle-associated risk factors and improvements in diagnostics. For example, proportion of overweight women increased from 42.9% in 1997 to 58.2% in 2003, the average age at first birth increased from 23.5 years in 1960 to 27.4 years in 2009, and the fertility rate decreased from 2.9 in 1950 to 1.5 in 2009 (34,35). Also, the increase may have been affected by a higher prevalence of alcohol consumption and the use of hormonal replacement therapy and oral contraceptives. The use of hormonal replacement therapy and oral contraceptives in Croatia is lower than in most European countries, but average alcohol intake is among the highest in Europe (36-38).

Croatian national screening program for early detection of breast cancer was introduced in 2006. It includes women aged 50-69 years. In the first screening cycle, 720 982 women were invited to mammography screening, with the response of 63% and more than 1500 detected breast cancers (39). The national screening program could have affected the increase in the incidence that appeared in the age group 60-69 years after 2005. However, the lack of similar increase in the age group 50-59 years does not confirm this hypothesis.

Besides its influence on the incidence, the national breast cancer screening program "Mamma" is also expected to reduce mortality. Countries with long-standing breast cancer screening program had reduced mortality in the screened population (40-42). However, the screening-related reduction in mortality is expected to occur in Croatia in the next 5-10 years (40-42).

Interestingly, in some countries the decline in breast cancer mortality had started before screening was introduced and occurred in non-screened population. Also, declines in mortality were also observed in countries without national screening programs (42). Similarly, the highest decline in mortality in our study was found in younger age groups. Also the decrease in mortality in the age-group 50-59 years started in 1999, before the introduction of the screening program, which could probably be attributed to improved breast cancer care.

### Endometrial and ovarian cancer

Gynecological cancers in different European countries have very different incidence trends. A study of geographic and temporal variations of endometrial cancer incidence in 13 European countries in the period from 1964 to 2000 (43) found increasing trends in Iceland, the Netherlands, Czech Republic, Slovakia, Slovenia, Finland, Norway, Sweden, United Kingdom, Spain, and Poland with EAPCs from +0.8 to +3.4% for menopausal women. Decreasing incidence trends were reported for Italy, Switzerland, and Denmark, with EAPCs from -0.8 to -1.1%, and Germany had stable incidence. The increasing incidence trend of endometrial cancer in Croatia with EAPC of 0.8% is similar to most of the European countries.

According to GLOBOCAN 2008 estimates, Croatia is among the 20 countries in Europe with the highest incidence of ovarian cancer (32). The countries that had a high incidence of ovarian cancer in 1960s and 1970s, like Nordic countries, Austria, Germany, and United Kingdom, nowadays show a declining incidence, while Southern and Eastern European countries show an increasing incidence (44). Our trend analysis for Croatia showed a stable incidence trend, with AAPC of 0.1%.

Increasing endometrial cancer incidence and high ovarian cancer incidence in Croatia could be explained by an increasing prevalence of factors such as obesity and low parity. Growing obesity is an important public health care problem. A recent study reported that 34% Croatian women are overweight (45). Another risk factor for endometrial cancer is diabetes mellitus, the total prevalence of which in adults in Croatia is high (8.9%) and growing (46). At the same time, oral contraception, which was associated with a decreased risk for endometrial and ovarian cancer, is rarely used (37). However, a significant decrease in the incidence of ovarian cancer in the period 2000-2008 could possibly be explained by an increased use of oral contraception since 1980s, but there are no scientific data to corroborate it. This decreasing incidence trend has not yet reflected on mortality, which has had a significantly increasing trend since 1992, with EAPC of 1.2%, in contrast to the trends observed in most of the other European populations (44). Also, the ASR remains high, placing Croatia among the countries with the highest ovarian cancer mortality in Europe (32).

### Cervical cancer

According to GLOBOCAN 2008 estimates, Croatia has an intermediate ASR of cervical cancer (32). From the middle 1990s to early 2000s, the rates of cervical cancer were highest in Central Europe, the lowest in Finland, Italy, and Malta, and stable in Nordic countries, Ireland, Austria, and the Netherlands (47). The largest decrease in incidence was observed in countries with organized screening programs like Finland and the Netherlands (48).

The introduction of the Pap smear, which detects premalignant lesions of the cervix and early stages of cervical cancer, reduced the incidence and mortality in developed countries (49). In Croatia, Pap smears were introduced in 1950s (50) and became part of the routine gynecological examination. This led to a decrease in incidence, but an even greater decrease could be achieved through an organized screening program. The Finnish model of organized Pap screening program shows to which extent it is possible to decrease cervical cancer incidence (51). In addition to an 80% reduction in cervical cancer incidence in the age-group 30-64 years, which can be achieved by organized screening (51), further decrease could be achieved by the use of HPV vaccines against oncogenic types (52). HPV vaccines have been available in Croatia since 2007, but vaccination is not obligatory (53). HPV vaccines cannot replace cytological screening for cervical cancer and their influence on cervical cancer incidence is yet to be seen (54). Croatia already has screening programs for breast and colorectal cancers (39), and the introduction of a screening program for cervical cancer is planned for the near future. However, more investment in cancer prevention is needed, in terms of reducing the exposure to highly prevalent lifestyle risk factors and maintaining or introducing high-quality national screening programs for breast and cervical cancers.
